# Ultraviolet irradiation increases free apigenin (aglycone) accumulation in parsley under controlled environments

**DOI:** 10.3389/fpls.2026.1975585

**Published:** 2026-10-21

**Authors:** Rebekah C. I. Maynard, Samuel O. Ogundipe, Rhuanito Soranz Ferrarezi, Joon Hyuk Suh, Leonardo Lombardini

**Affiliations:** 1 Department of Biology, University of North Georgia, Dahlonega, Georgia; 2 Department of Food Science and Technology, University of Georgia, Athens, Georgia; 3 Department of Horticulture, University of Georgia, Athens, Georgia

**Keywords:** anticancer, apigenin, growth chamber, light intensity, ultraviolet

## Abstract

**Introduction:**

Parsley (*Petroselinum crispum*) is a productive natural source of apigenin, a medicinally valuable secondary metabolite. This study evaluated the effects of light intensity and ultraviolet (UV) exposure on free apigenin (aglycone) accumulation in the Giant of Italy parsley cultivar grown under controlled environment conditions.

**Methods:**

Plants were grown under light-emitting diodes (LED) with a 16-h photoperiod and daily light integrals (DLI) of 19.0, 23.8, or 28.5 mol m^-2^ d^-1^. After eight weeks, mature leaves from control plants were harvested for analysis. The remaining plants received supplemental UV light at 475 μ cm^-2^ to serve as a stressor for inducing free apigenin biosynthesis throughout the 16-h photoperiod for 1, 2, 4, or 8 days. Free apigenin was quantified in lyophilized leaf samples with high-performance liquid chromatography–ultraviolet (HPLC–UV) detection.

**Results:**

LED light intensity did not affect free apigenin accumulation, whereas UV exposure significantly increased it. The 8-d UV treatment produced the highest free apigenin content, at 10.44 ± 1.91 mg kg^-1^, compared with 1.08 ± 0.31 mg kg^-1^ in the untreated control, representing an 867% increase.

**Discussion:**

These findings suggest that supplemental UV exposure may be an effective strategy for increasing free apigenin accumulation in parsley grown under controlled environmental conditions. Among the exposure durations tested, the greatest accumulation occurred after eight days of UV treatment.

## 1 Introduction

Plants are naturally exposed to a broad range of solar radiation, including ultraviolet B (UV-B) (290-315 nm), ultraviolet A (UV-A) (315-400 nm), visible (400-750 nm), and infrared (>750 nm) light. Approximately 5% of the solar radiation transmitted through Earth’s atmosphere is in the (UV) range, and due to the high absorption by ozone, only 0.25% is UV-B (IARC, 2012). Plants detect UV-B light with the UV RESISTANCE LOCUS 8 (UVR8) protein (Christie et al., 2012) and exhibit protective responses such as reducing cell expansion (Fasano et al., 2014), suppressing hypocotyl growth (Cloix et al., 2012), and synthesizing flavonoids to absorb the UV radiation (Koes et al., 1994). Plants are adapted to respond to the levels of UV in solar radiation; however, controlled environment experiments have indicated that elevated UV-B can damage plant DNA, cause organelle dysfunction, and ultimately lead to cell death (Nawkar et al., 2013).

UV-B light is not considered essential for plant survival. It has been demonstrated that plants, such as chrysanthemum (*Chrysanthemum × gradiflorum*) (Kim et al., 2004), lettuce (*Lactuca sativa*) (Chen et al., 2019), and strawberry (*Fragaria × ananassa*) (Samuolienė et al., 2010), can be grown in controlled environments using blue and red light-emitting diodes (LEDs) without UV light. Nevertheless, the addition of UV light to controlled environments is a useful method of increasing the biosynthetic production of flavonoids. For example, treatment with UV-B light increased quercetin and luteolin in European privet (*Ligustrum vulgare*) (Agati et al., 2011), kaempferol in *Petunia* (Ryan et al., 2002), and isorhamnetin in ginkgo (*Ginkgo biloba*) (Zhao et al., 2020).

One important flavonoid, apigenin, is biosynthesized in plants to protect against UV-B damage (Righini et al., 2019) by strongly absorbing in the UV range (Amat et al., 2009). Furthermore, apigenin accumulation is enhanced with exposure to UV-A (Fan et al., 2025; Li et al., 2025). Apigenin is medicinally significant with effectiveness against bladder (Zhu et al., 2013), breast (Pham et al., 2021), cervical (Chen et al., 2022), colorectal (Cheng et al., 2021), and prostate (Costea et al., 2020) cancers. It is also useful as an antifungal (Singh et al., 2014), anti-inflammatory (Wang et al., 2014), and antioxidant agent (Tian et al., 2021).

Apigenin can be chemically synthesized for medicinal use, but the process is multi-day and low-yielding (Poureini et al., 2022). Alternatively, plants, such as parsley, have been recognized as important natural sources of apigenin. Although much of the apigenin in parsley occurs as glycosylated derivatives, free apigenin aglycone is also present and can be directly extracted and quantified without prior hydrolysis (Boldizsár et al., 2013; Michalaki et al., 2023; Ibrahiem et al., 2025). The concentration of free apigenin reported in parsley varies considerably depending on cultivar, growing conditions, and extraction method. For example, direct ethanolic extraction of dried parsley without hydrolysis has yielded free apigenin concentrations of approximately 4.3–21.0 mg kg^-^¹ dry weight, demonstrating that naturally occurring free apigenin represents a relatively small but measurable fraction of the apigenin present in parsley (Michalaki et al., 2023). In contrast, substantially higher values (7,900–9,480 mg kg^-^¹ dry weight), have been reported when analytical procedures include conditions that measure a broader apigenin pool (Wang et al., 2015; Poureini et al., 2020).

Because of its medicinal value, apigenin may be a promising target for enhanced biosynthetic production in plants. One possible method to increase apigenin accumulation is to expose plants to higher levels of UV radiation. Elevated apigenin levels in celery (*Apium graveolens*) were correlated with increased transcription of the chalcone synthase (CHS), chalcone isomerase (CHI), and flavone synthase (FSI) enzymes (Yan et al., 2014), which are directly involved in the biosynthetic pathway of apigenin (Marín et al., 2017). Furthermore, CHS activity increased in response to supplemental UV irradiation in parsley (Schmelzer et al., 1988). Based on these findings, we hypothesize that free apigenin will accumulate at higher levels in plants through exposure to UV light. Therefore, the aim of this study was to determine the impact of increasing LED-generated light intensity and UV light on free apigenin production in parsley.

## 2 Materials and methods

### 2.1 Plant material and environmental conditions

Parsley ‘Giant of Italy’ seeds (Johnny’s Selected Seeds, Winslow, ME) were individually sown on substrate (PRO-MIX HP; Premier Tech Horticulture, Quakertown, PA) in 96-well trays in three identical 1.8 × 0.8 m growth chambers (CMP 3244; Conviron, Pembina, ND) located at the University of Georgia (College of Agricultural and Environmental Sciences, Controlled Environment Agriculture Crop Physiology and Production Laboratory). The temperature in each growth chamber was maintained at 24 °C during the day and 20 °C at night with a 16-h photoperiod, and the trays were manually irrigated overhead to maintain a consistently moist substrate.

### 2.2 Treatment and experimental design

The daily light integral (DLI) was set differently in the three growth chambers to determine the impact of supplemental light-emitting diode (LED) light intensity on free apigenin accumulation. The LED lights were sole-source panels (P1000-300-02; ViparSpectra, Richmond, CA) with 600 nm red, 3000 K – 5000 K white, and 730 nm far-red. One growth chamber received 19.0 mol m^-2^ d^-1^ of light as recommended by Litvin-Zabal (2019), and the other two growth chambers had a 25% and 50% increase in the recommended DLI with 23.8 and 28.5 mol m^-2^ d^-1^, respectively. After five weeks, the seedlings were transplanted into 5-cm pots, and after two more weeks, into 10-cm pots. The plants were arranged in a randomized complete block design within each growth chamber based on their assigned light treatment. Each growth chamber contained eight plants that received 0, 1, 2, 4, or 8 days of exposure to ultraviolet (UV) light.

### 2.3 Pre-treatment harvesting and UV light application

The control plants, receiving 0 days of UV light, were harvested eight weeks after sowing at the end of the 8-h dark period. Due to the size of the plants at harvest, two plants were combined per replicate, with four replicates per treatment per growth chamber. The fully expanded parsley leaves were collected into screw-top tubes (Falcon; Corning Inc., Corning, NY) and transferred to a Dewar flask containing liquid nitrogen. Each sample was dried at -84 °C and 0.133 mbar for 24 h in a lyophilizer (Labconco; Marshall Scientific, Hampton, NH). Then, the freeze-dried samples were ground into a fine powder using a mortar and pestle and stored at -80 °C.

All remaining plants in the experiment were left in the growth chambers to receive 1, 2, 4, or 8 days of UV light. The LED light was supplemented with UV light provided by 18W fluorescent tubes (MIGRO UVB 310; MIGRO Grow Lights, Dublin, Ireland) consisting of 25% UV-A and 75% UV-B light with the peak spectral output at 310 nm. Three light tubes were spaced 0.6 m apart in the growth chambers. The average 310 nm light intensity, measured at the height of the pots with a light meter (Light Meter UVA/B; Sper Scientific Direct, Scottsdale, AZ), was 475 μW cm^-2^. The LED light was supplemented with UV light throughout the 16-h photoperiod each day, providing 273.6 kJ m^-2^ d^-1^ of UV. The UV-treated parsley plants were left in the dark for 8 h before the leaves were harvested, following the procedure described for the control. The method of harvesting after a dark period was based on a previous study of parsley, which found that mRNA levels of the CHS enzyme, which is part of the apigenin biosynthesis pathway, increased during a 16-h photoperiod of UV-containing light and continued to increase through an 8-h dark period following the treatment (Schmelzer et al., 1988).

### 2.4 Free apigenin quantification

The dried leaf samples were analyzed using high-performance liquid chromatography–photodiode array (HPLC–PDA) detection to quantify free apigenin in the tissue. A stock solution was prepared at a concentration of 1 mg mL^-1^ by dissolving the apigenin standard (95% purity) in HPLC-grade methanol/DMSO (90/10, v/v) (Sigma-Aldrich, St. Louis, MO). The working standard solutions (calibration standards) were prepared daily by diluting the stock solution with methanol prior to use. The lyophilized parsley leaves (100 mg) were mixed with 1 mL of 50% methanol in 2 mL plastic tubes. The samples were vortexed for 10 min at 4 °C and centrifuged at 13,500 × g for 10 min. The supernatants were collected, passed through 0.2 µm membrane filters, and injected into the HPLC system.

The HPLC (Shimadzu Nexera; Shimadzu Corp., Tokyo, Japan) used for apigenin quantification was equipped with a photodiode array detector. Apigenin was separated on a 4.6 × 150 mm analytical column with a 5 μm particle size equipped with a guard column (ZORBAX Eclipse XDB-C18; Agilent Technologies, Santa Clara, CA). The column temperature was set to 35 °C. The mobile phase was composed of water/acetonitrile (65/35, v/v, %) containing 0.1% formic acid (Oakwood Products Inc., Estill, SC). The flow rate was 1 mL min^-1^ with an injection volume of 5 μL. After chromatographic separation, apigenin was detected at a UV wavelength of 336 nm and identified by comparing the retention times and UV spectra with the apigenin standard. Apigenin was quantified using standard calibration curves. The limits of detection (LOD) and quantification (LOQ) were calculated as 3.3σ/S and 10σ/S, respectively. Extraction recovery (n = 3) was evaluated by spiking parsley samples with the apigenin standard at low (1.0 µg mL^-^¹), medium (4.0 µg mL^-^¹), and high (8.0 µg mL^-^¹) levels, and recovery was expressed as the percentage of added apigenin recovered.

### 2.5 Experimental design and statistical analysis

The experiment was set up as a two-way factorial with a randomized complete block design. The first factor, with three levels, was LED light intensity. The second factor, with five levels, was days of UV exposure. Each treatment group contained eight parsley plants. LabSolutions software (Ver. 5.124) was used for the HPLC data interpretation. RStudio (version 2023.12.0 + 369) was used for statistical analysis.

## 3 Results and discussion

### 3.1 HPLC method evaluation

Apigenin was clearly separated from matrices (**Figure 1**), with a retention time of 5.4 min. No matrix interference was observed at the retention time of apigenin, demonstrating adequate selectivity of the HPLC method for apigenin determination in parsley extracts. Linearity was evaluated over an apigenin concentration range of 0.1 to 10.0 µg mL^-^¹. The calibration curve showed excellent linearity and was described by the regression equation y = 5887x + 33, with a coefficient of determination (r²) of 0.9999. The method showed a limit of detection (LOD) of 0.0083 µg mL^-^¹ and a limit of quantification (LOQ) of 0.0252 µg mL^-^¹. Extraction recovery ranged from 94.6 to 101.3%, with a mean recovery of 97.93 ± 3.35%, indicating efficient recovery of free apigenin from the parsley matrix. Therefore, HPLC analysis was considered an appropriate method for isolating and quantifying apigenin abundance.

**Figure 1:**
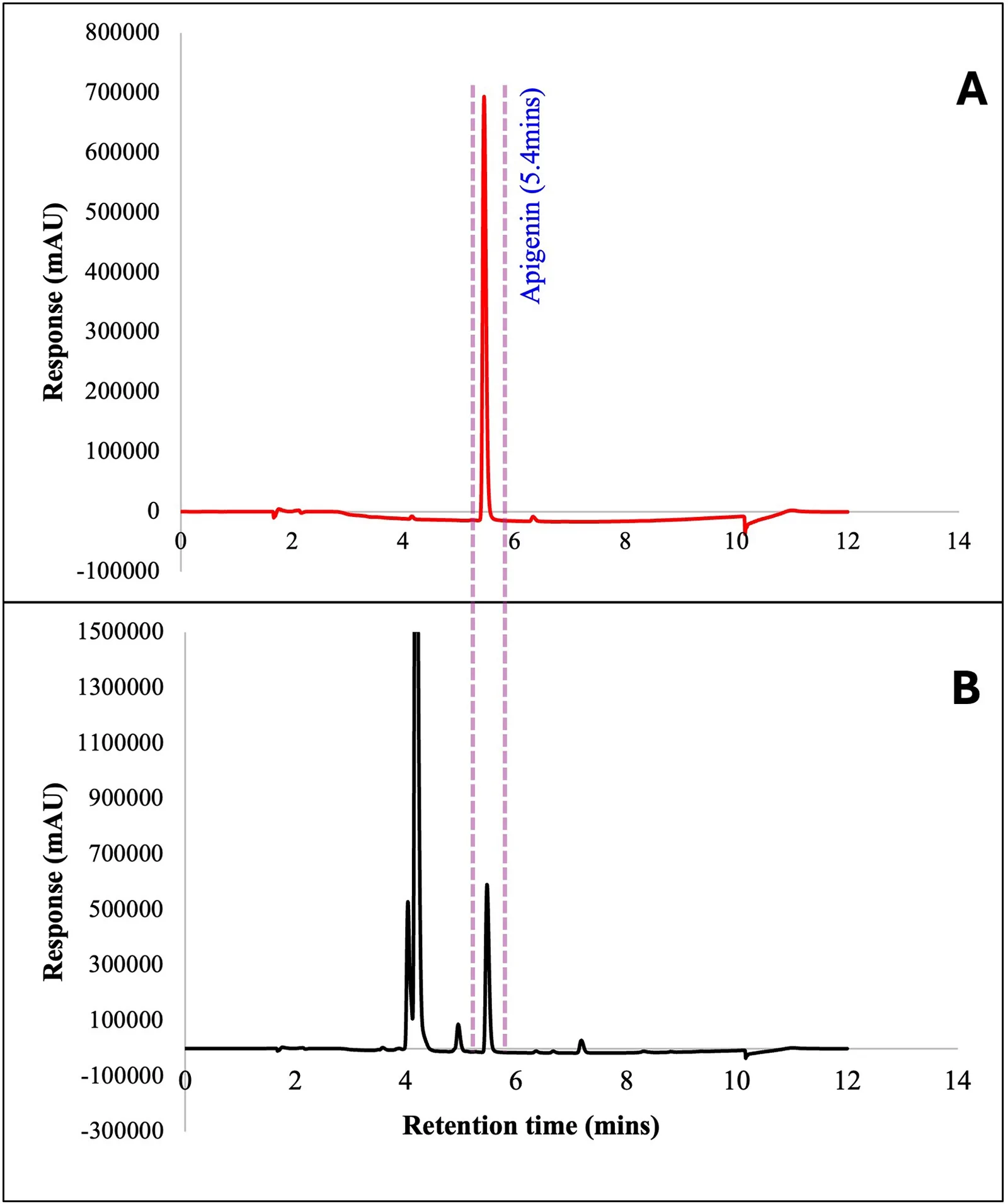
HPLC chromatograms of free apigenin from the standard **(A)** and a sample of lyophilized parsley leaves **(B)**.

### 3.2 Free apigenin accumulation in response to LED intensity

One objective of this study was to investigate the impact of high-intensity light under the normal photosynthetically active range on free apigenin accumulation in parsley. Across the treatment groups, free apigenin was detected in the range of 0.61-16.52 mg kg^-1^ (**Figure 2**). Although there appeared to be a trend of lower free apigenin accumulation at higher LED intensities, the differences were not statistically significant (F(2, 45) = 2.67, p = 0.081). Therefore, the LED intensities used in this study did not impact free apigenin accumulation.

**Figure 2:**
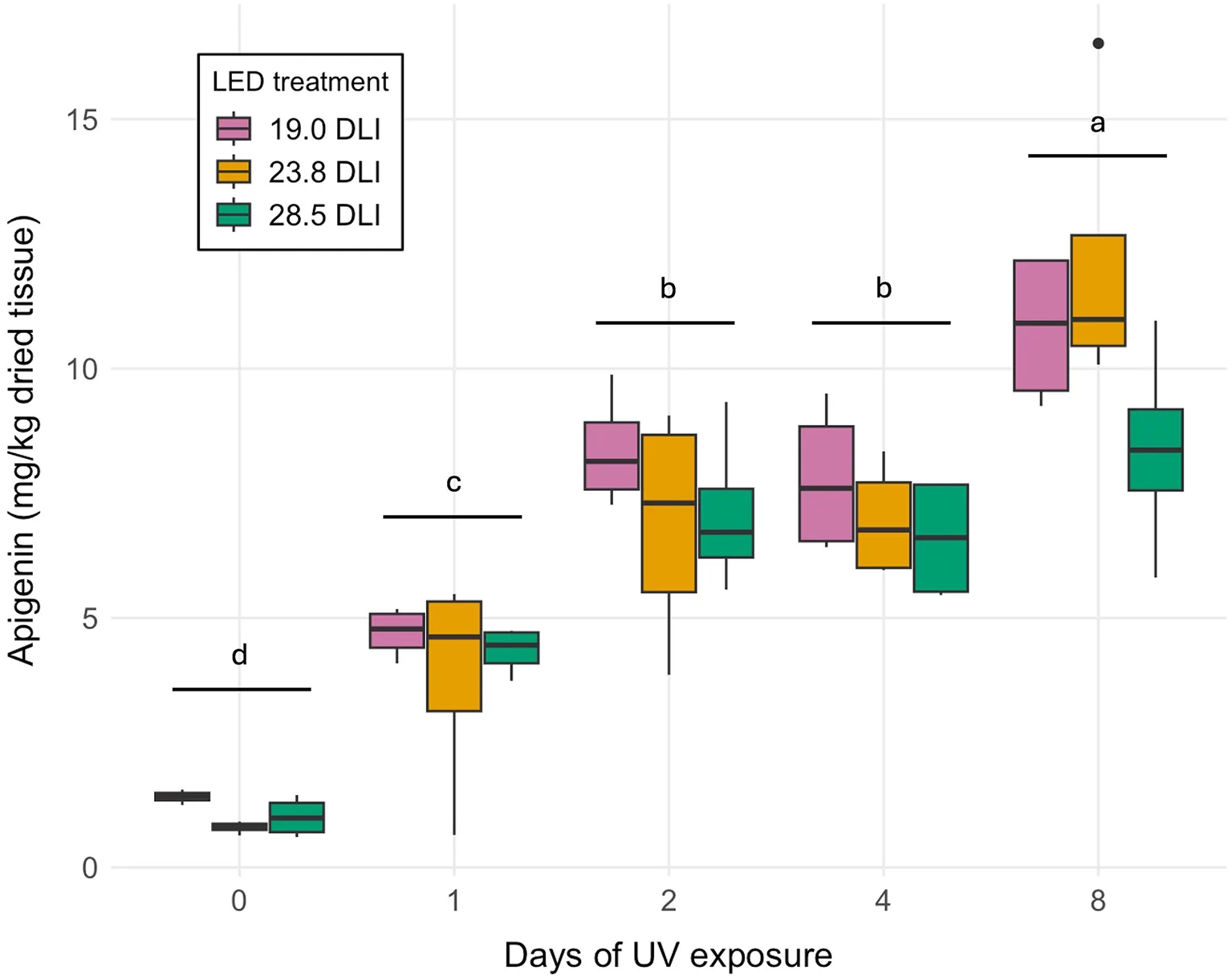
Free apigenin accumulation was quantified in dried parsley leaves following different light treatments. The parsley plants were exposed to 19.0, 23.8, or 28.5 DLI of LED light and up to eight days of UV light. Different letters indicate statistically significant differences among treatment means based on Tukey’s HSD test (p < 0.05).

Because the free apigenin levels were not significantly different in parsley under different LED light intensities, the data collected across the three growth chambers was averaged (**Table 1**).

**Table 1:** Concentration of free apigenin in dried parsley leaves under different LED and UV light treatments.

UV	LED	Free Apigenin
(d)	(mol m^-2^ d^-1^)	(mg kg^-1^ dried tissue)
0	19	1.42 ± 0.13
	23.8	0.80 ± 0.12
	28.5	1.01 ± 0.40
	Mean	1.08 ± 0.31^d^
1	19	4.71 ± 0.50
	23.8	3.84 ± 2.23
	28.5	4.35 ± 0.47
	Mean	4.30 ± 0.43^c^
2	19	8.36 ± 1.16
	23.8	6.88 ± 2.40
	28.5	7.09 ± 1.61
	Mean	7.44 ± 0.63^b^
4	19	7.78 ± 1.52
	23.8	6.96 ± 1.17
	28.5	6.59 ± 1.25
	Mean	7.11 ± 0.61^b^
8	19	10.81 ± 1.58
	23.8	12.14 ± 2.97
	28.5	8.38 ± 2.11
	Mean	10.44 ± 1.91^a^
		p-values
UV		<0.001
LED		0.081
UV × LED		0.233

Values are reported as the mean ± standard deviation. Different letters indicate statistically significant differences among treatment means based on Tukey’s HSD test (p < 0.05).

In previous research, sweet basil (*Ocimum basilicum*) grown below the recommended DLI resulted in lower total flavonoid content (Dou et al., 2018). Furthermore, a study of strawberry (*Fragaria × ananassa*) found that flavonoid accumulation decreased at elevated light levels (Maeda and Ito, 2020). These studies suggest that flavonoid accumulation can be negatively affected at DLI levels above or below the recommended level. The present study exceeded the recommended light intensity for parsley by a 25% and 50% increase. No statistically significant positive or negative impact was measured on free apigenin accumulation. However, it is possible that DLI beyond the range used in this experiment could impact free apigenin accumulation in parsley.

### 3.3 Free apigenin accumulation in response to UV exposure

The impact of UV light on free apigenin accumulation was statistically significant (F(4, 45) = 62.11, p < 0.001). Free apigenin accumulation increased with prolonged UV exposure; however, there was no significant difference between apigenin accumulation between day 2 and day 4. The greatest free apigenin accumulation occurred after 8 d of UV exposure with 10.44 ± 1.91 mg kg^-1^ per dried parsley leaves. Compared with the control (1.08 ± 0.31 mg kg^-1^ dried tissue), the 8-d UV exposure resulted in an 867% increase in free apigenin concentration. In contrast, a study of hairy root cultures of pigeon pea (*Cajanus cajan*) found the greatest concentration of free apigenin, approximately 7 mg kg^-1^ dried tissue, accumulated after 4 h of irradiation with UV light and gradually decreased with longer exposure (Gai et al., 2022). Although the present study found an increase in free apigenin through 8 d of UV exposure, it is not known whether free apigenin would have a response to reduced or prolonged treatment.

Because the experiment was conducted in growth chambers with no UV output from the LED lights, the control plants did not receive any UV light prior to harvest. However, free apigenin was detected in the control parsley, albeit at very low levels. Many studies report the positive influence of UV irradiation on flavonoid accumulation (Agati et al., 2011; Ryan et al., 2002; Zhao et al., 2020), but our findings show that UV light is not essential for apigenin biosynthesis. Although free apigenin accumulated in the absence of UV light, its concentration increased significantly with greater UV exposure in the treated groups (F(4, 45) = 62.11, p < 0.001).

### 3.4 Interaction of LED and UV exposure on free apigenin accumulation

There was no significant interaction between LED light intensity and UV exposure on free apigenin accumulation in parsley (F(8, 45) = 1.38, p = 0.233). Therefore, UV light alone had a strong positive impact on free apigenin accumulation. Although the impact of LED light intensity was not statistically significant, the slight variation in free apigenin accumulation across the three LED treatment groups may be due to the proportion of UV in the overall light source. The parsley plants grown at the recommended DLI received a light intensity of 330 μmol m^-2^ s^-1^ from the LED lights, with the higher LED light treatments receiving 412 and 495 μmol m^-2^ s^-1^, respectively. The intensity of UV light was constant across the three growth chambers, meaning that at higher LED intensities, a lower percentage of the overall light was UV. At the highest LED intensity, UV accounted for approximately 2.5% of the total radiation, whereas at the lowest LED intensity, UV accounted for approximately 3.7% of the total radiation. Even though the percentage of UV light varied across growth chambers, the treatment groups still experienced a six to nine-fold increase in UV intensity compared to the 0.25% in normal daylight (IARC, 2012). Therefore, the plants in this study experienced higher levels of UV light than they would in field conditions. Despite the elevated UV levels, there was no negative impact on the plant’s appearance based on visual observation.

## 4 Conclusion

Apigenin is an effective biopharmaceutical and may be naturally increased in plants by manipulating the wavelength range and intensity of the light source. The present study determined that free apigenin accumulation was not impacted by exceeding the recommended DLI with LED light. Furthermore, UV light is not essential for the biosynthesis of free apigenin since free apigenin was measured in parsley grown without UV light. However, free apigenin accumulation increased with longer exposure to UV. The treatment group with 8 d of UV light had the highest free apigenin concentration with 10.44 ± 1.91 mg kg^-1^ dried tissue, which was 867% greater than the control group. Because free apigenin accumulation was not impacted by high DLIs applied by LED lights, future research should focus on using a higher-intensity UV source and prolonging exposure beyond the 8-d period to determine the optimal UV light treatment for maximizing free apigenin production in parsley. In addition, the extent to which apigenin glycosides accumulate under these conditions remains to be elucidated, and quantification of total apigenin following hydrolysis is needed to establish absolute production potential.

## Data Availability

The datasets presented in this study can be found in online repositories. The names of the repository/repositories and accession number(s) can be found in the article/supplementary material.

## References

[B1] Agati G. Biricolti S. Guidi L. Ferrini F. Fini A. Tattini M. (2011). The biosynthesis of flavonoids is enhanced similarly by UV radiation and root zone salinity in *L. vulgare* leaves. J. Plant Physiol. 168, 204–212. doi: 10.1016/j.jplph.2010.07.016 20850892

[B2] Amat A. Clementi C. De Angelis F. Sgamellotti A. Fantacci S. (2009). Absorption and emission of the apigenin and luteolin flavonoids: A TDDFT investigation. J. Phys. Chem. A 113, 15118–15126. doi: 10.1021/jp9052538 19722543

[B3] Boldizsár I. Füzfai Z. Molnár-Perl I. (2013). Characterization of the endogenous enzymatic hydrolyses of *Petroselinum crispum* glycosides: Determined by chromatography upon their sugar and flavonoid products. J. Chromatogr. A 1293, 100–106. doi: 10.1016/j.chroma.2013.03.037 23623367

[B4] Chen X. L. Wang L. C. Li T. Yang Q. C. Guo W. Z. (2019). Sugar accumulation and growth of lettuce exposed to different lighting modes of red and blue LED light. Sci. Rep. 9, 6926. doi: 10.1038/s41598-019-43498-8 31061448 PMC6502839

[B5] Chen Y. H. Wu J. X. Yang S. F. Yang C. K. Chen T. H. Hsiao Y. H. (2022). Anticancer effects and molecular mechanisms of apigenin in cervical cancer cells. Cancers 14, 1824. doi: 10.3390/cancers14071824 35406599 PMC8998024

[B6] Cheng Y. Han X. Mo F. Zeng H. Zhao Y. Wang H. . (2021). Apigenin inhibits the growth of colorectal cancer through down-regulation of E2F1/3 by miRNA-215-5p. Phytomedicine 89, 153603. doi: 10.1016/j.phymed.2021.153603 34175590

[B7] Christie J. M. Arvai A. S. Baxter K. J. Heilmann M. Pratt A. J. O’Hara A. . (2012). Plant UVR8 photoreceptor senses UV-B by tryptophan-mediated disruption of cross-dimer salt bridges. Science 335, 1492–1496. doi: 10.1126/science.1218091 22323738 PMC3505452

[B8] Cloix C. Kaiserli E. Heilmann M. Baxter K. J. Brown B. A. O’Hara A. . (2012). C-terminal region of the UV-B photoreceptor UVR8 initiates signaling through interaction with the COP1 protein. Proc. Natl. Acad. Sci. 109, 16366–16370. doi: 10.1073/pnas.1210898109 22988111 PMC3479605

[B9] Costea T. Vlad O. C. Miclea L. C. Ganea C. Szöllősi J. Mocanu M. M. (2020). Alleviation of multidrug resistance by flavonoid and non-flavonoid compounds in breast, lung, colorectal and prostate cancer. Int. J. Mol. Sci. 21, 401. doi: 10.3390/ijms21020401 31936346 PMC7013436

[B10] Dou H. Niu G. Gu M. Masabni J. G. (2018). Responses of sweet basil to different daily light integrals in photosynthesis, morphology, yield, and nutritional quality. HortScience 53, 496–503. doi: 10.21273/hortsci12785-17

[B11] Fan Y. Huang W. Gao H. Zhang S. Zhang X. Zhao J. . (2025). A novel strategy of using ultraviolet a light emitting diodes (UVA-LED) irradiation extends the shelf-life and enhances the antioxidant property of minimally processed pakchoi (*Brassica rapa* subsp. *chinensis*). Food Chem. 29, 102826. doi: 10.1016/j.fochx.2025.102826 40756335 PMC12318348

[B12] Fasano R. Gonzalez N. Tosco A. Dal Piaz F. Docimo T. Serrano R. . (2014). Role of Arabidopsis UV RESISTANCE LOCUS 8 in plant growth reduction under osmotic stress and low levels of UV-B. Mol. Plant 7, 773–791. doi: 10.1093/mp/ssu002 24413416

[B13] Gai Q. Y. Lu Y. Jiao J. Fu J. X. Xu X. J. Yao L. . (2022). Application of UV-B radiation for enhancing the accumulation of bioactive phenolic compounds in pigeon pea [*Cajanus cajan* (L.) Millsp.] hairy root cultures. J. Phytochem. Phytobiol. B. 228, 112406. doi: 10.1016/j.jphotobiol.2022.112406 35152064

[B14] IARC (2012). “ Radiation: A review of human carcinogens,” in Solar and Ultraviolet Radiation (Lyon (FR): International Agency for Research on Cancer), 35–101.

[B15] Ibrahiem A. A. Mosa A. A. Mohammed L. Y. (2025). Polyphenol profile, antioxidant potential, vitamin C and mineral contents of crude parsley leaves extract (*Petroselinum crispum*) and its isolated apigenin: A comparative study. Baghdad Sci. J. 22, 1465–1476. doi: 10.21123/bsj.2024.9622

[B16] Kim S. J. Hahn E. J. Heo J. W. Paek K. Y. (2004). Effects of LEDs on net photosynthetic rate, growth and leaf stomata of chrysanthemum plantlets *in vitro*. Sci. Hortic. 101, 143–151. doi: 10.1016/j.scienta.2003.10.00342574925

[B17] Koes R. E. Quattrocchio F. Mol J. N. M. (1994). The flavonoid biosynthetic pathway in plants: Function and evolution. BioEssays 16, 123–132. doi: 10.1002/bies.95016020942587375

[B18] Li D. Shen H. Yu L. Zhu K. Zhang Y. Wu S. . (2025). Transcriptomic and metabolomic insights into plant hormone modulation and secondary metabolite accumulation in basil under red and ultravioolet-A light. Ing. J. Mol. Sci. 26, 3758. doi: 10.3390/ijms26083758 40332413 PMC12027552

[B19] Litvin-Zabal A. G. (2019). “ Quantifying the effects of light quantity and quality on culinary herb physiology PhD Diss,” Quantifying the effects of light quantity and quality on culinary herb physiology. ( Iowa State University, Ames, IA).

[B20] Maeda K. Ito Y. (2020). Effect of different PPFDs and photoperiods on growth and yield of everbearing strawberry ‘Elan’ in plant factory with white LED lighting. Environ. Contr. Biol. 58, 99–104. doi: 10.2525/ecb.58.99

[B21] Marín L. Gutiérrez-del-Río I. Yagüe P. Manteca Á. Villar C. J. Lombó F. (2017). De novo biosynthesis of apigenin, luteolin, and eriodictyol in the actinomycete *Streptomyces albus* and production improvement by feeding and spore conditioning. Front. Microbiol. 8. doi: 10.3389/fmicb.2017.00921 28611737 PMC5447737

[B22] Michalaki A. Karantonis H. C. Kritikou A. S. Thomaidis N. S. Dasenaki M. E. (2023). Ultrasound-assisted extraction of specific phenolic compounds from *Petroselinum crispum* leaves using response surface methodology and HPLC-PDA and Q-TOF-MS/MS identification. Appl. Sci. 13, 798. doi: 10.3390/app13020798 36903279 PMC10004109

[B23] Nawkar G. M. Maibam P. Park J. H. Sahi V. P. Lee S. Y. Kang C. H. (2013). UV-induced cell death in plants. Int. J. Mol. Sci. 14, 1608–1628. doi: 10.3390/ijms14011608 23344059 PMC3565337

[B24] Pham T. H. Page Y. L. Percevault F. Ferrière F. Flouriot G. Pakdel F. (2021). Apigenin, a partial antagonist of the estrogen receptor (ER), inhibits ER-positive breast cancer cell proliferation through Akt/FOXM1 signaling. Int. J. Mol. Sci. 22, 470. doi: 10.3390/ijms22010470 33466512 PMC7796491

[B25] Poureini F. Mohammadi M. Najafpour G. D. Nikzad M. (2020). Comparative study on the extraction of apigenin from parsley leaves (*Petroselinum crispum* L.) by ultrasonic and microwave methods. Chem. Pap. 74, 3857–3871. doi: 10.1007/s11696-020-01208-z30311153

[B26] Poureini F. Najafpour G. Nikzad M. Mohammadi M. (2022). Antioxidant activity of parsley leaves extract containing apigenin obtained by ultrasonic extraction. J. Nat. Compd. Chem. 1, 1–6. doi: 10.22092/JNCC.2023.129055

[B27] Righini S. Rodriguez E. J. Berosich C. Grotewold E. Casati P. Falcone Ferreyra M. L. (2019). Apigenin produced by maize flavone synthase I and II protects plants against UV-B-induced damage. Plant Cell Environ. 42, 495–508. doi: 10.1111/pce.13428 30160312

[B28] Ryan K. G. Swinny E. E. Markham K. R. Winefield C. (2002). Flavonoid gene expression and UV photoprotection in transgenic and mutant *Petunia* leaves. Phytochemistry 59, 23–32. doi: 10.1016/S0031-9422(01)00404-6 11754940

[B29] Samuolienė G. Brazaitytė A. Urbonavičiūtė A. Šabajevienė G. Duchovskis P. (2010). The effect of red and blue light component on the growth and development of frigo strawberries. Zemdirbyste 97, 99–104.

[B30] Schmelzer E. Jahnen W. Hahlbrock K. (1988). *In situ* localization of light-induced chalcone synthase mRNA, chalcone synthase, and flavonoid end products in epidermal cells of parsley leaves. Proc. Natl. Acad. Sci. 85, 2989–2993. doi: 10.1073/pnas.85.9.2989 16578833 PMC280128

[B31] Singh G. Kumar P. Joshi S. C. (2014). Treatment of dermatophytosis by a new antifungal agent ‘apigenin’. Mycoses 57, 497–506. doi: 10.1111/myc.12188 24708558

[B32] Tian C. Liu X. Chang Y. Wang R. Lv T. Cui C. . (2021). Investigation of the anti-inflammatory and antioxidant activities of luteolin, kaempferol, apigenin and quercetin. S. Afr. J. Bot. 137, 257–264. doi: 10.1016/j.sajb.2020.10.02242574925

[B33] Wang J. Liu Y. T. Xiao L. Zhu L. Wang Q. Yan T. (2014). Anti-inflammatory effects of apigenin in lipopolysaccharide-induced inflammatory in acute lung injury by suppressing COX-2 and NF-kB pathway. Inflammation 37, 2085–2090. doi: 10.1007/s10753-014-9942-x 24958013

[B34] Wang Q. Cui W. Liu M. Zhang J. Liao R. Q. Liao X. L. . (2015). An improved synthesis of apigenin. J. Chem. Res. 39, 67–69. doi: 10.3184/174751915X14204548288464

[B35] Yan J. Yu L. Xu S. Gu W. Zhu W. (2014). Apigenin accumulation and expression analysis of apigenin biosynthesis relative genes in celery. Sci. Hortic. 165, 218–224. doi: 10.1016/j.scienta.2013.11.01842574925

[B36] Zhao B. Wang L. Pang S. Jia Z. Wang L. Li W. . (2020). UV-B promotes flavonoid synthesis in Ginkgo biloba leaves. Ind. Crop Prod. 151, 112483. doi: 10.1016/j.indcrop.2020.11248342574925

[B37] Zhu Y. Mao Y. Chen H. Lin Y. Hu Z. Wu J. . (2013). Apigenin promotes apoptosis, inhibits invasion and induces cell cycle arrest of T24 human bladder cancer cells. Cancer Cell Int. 13, 1–7. doi: 10.1186/1475-2867-13-54 23724790 PMC3674905

